# Diversity of Bacteria Associated with *Bursaphelenchus xylophilus* and Other Nematodes Isolated from *Pinus pinaster* Trees with Pine Wilt Disease

**DOI:** 10.1371/journal.pone.0015191

**Published:** 2010-12-09

**Authors:** Diogo Neves Proença, Romeu Francisco, Clara Vieira Santos, André Lopes, Luís Fonseca, Isabel M. O. Abrantes, Paula V. Morais

**Affiliations:** 1 Institute of Marine Research (IMAR-CMA), Coimbra, Portugal; 2 Department of Life Sciences, Faculty of Sciences and Technology (FCTUC), University of Coimbra, Coimbra, Portugal; Charité-University Medicine Berlin, Germany

## Abstract

The pinewood nematode (PWN), *Bursaphelenchus xylophilus*, has been thought to be the only causal agent of pine wilt disease (PWD), however, since bacteria have been suggested to play a role in PWD, it is important to know the diversity of the microbial community associated to it. This study aimed to assess the microbial community associated with *B. xylophilus* and with other nematodes isolated from pine trees, *Pinus pinaster*, with PWD from three different affected forest areas in Portugal. One hundred and twenty three bacteria strains were isolated from PWN and other nematodes collected from 14 *P. pinaster*. The bacteria strains were identified by comparative analysis of the 16S rRNA gene partial sequence. All except one Gram-positive strain (*Actinobacteria*) belonged to the Gram-negative *Beta* and *Gammaproteobacteria*. Most isolates belonged to the genus *Pseudomonas*, *Burkholderia* and to the family *Enterobacteriaceae*. Species isolated in higher percentage were *Pseudomonas lutea*, *Yersinia intermedia* and *Burkholderia tuberum*. The major bacterial population associated to the nematodes differed according to the forest area and none of the isolated bacterial species was found in all different forest areas. For each of the sampled areas, 60 to 100% of the isolates produced siderophores and at least 40% produced lipases. The ability to produce siderophores and lipases by most isolates enables these bacteria to have a role in plant physiological response. This research showed a high diversity of the microbial community associated with *B. xylophilus* and other nematodes isolated from *P. pinaster* with PWD.

## Introduction

The importance and potentially devastating impact of tree diseases was recognized early in the 20th century with severe epidemics associated with the introduction of new pathogens to native forest ecosystems. The pinewood nematode (PWN), *Bursaphelenchus xylophilus* [1] is the causal agent of the Pine Wilt Disease (PWD). Native to North-America, it was introduced to Japan and has spread into China, Korea and into Europe (Portugal and Spain) [2]. The etiology of the disease has not been well understood although PWN was confirmed to be the causative agent [3]. In general, browning of the tissues is caused by oxidation of phenols which occurs as a result of cellular disorganization [4], [5]. Since the physiological and histological changes in the diseased trees occur before a rapid increase in the number of nematodes, it is alleged that other participants might be involved in the pathological process [6]. Bacteria in association with *B. xylophilus* have been proposed to be needed for PWD development in *Pinus thunbergii* [7], but although studies were performed on isolates, none of the studies [7]–[12] included the molecular analysis of the microbial community associated with the nematode. Furthermore, the presence of bacteria in plant tissues (endophytes) is usually recognized as positive for the plant. The term endophyte is defined as ‘an organism inhabiting plant organs that at some time in its life can colonize internal plant tissue without causing apparent harm to the host’[13].

The objective of this study was to evaluate the diversity and stability of the bacterial community associated with *B. xylophylus* and other nematodes isolated from *P. pinaster* with PWD.

## Materials and Methods

### Sampling areas

**Figure 1:**
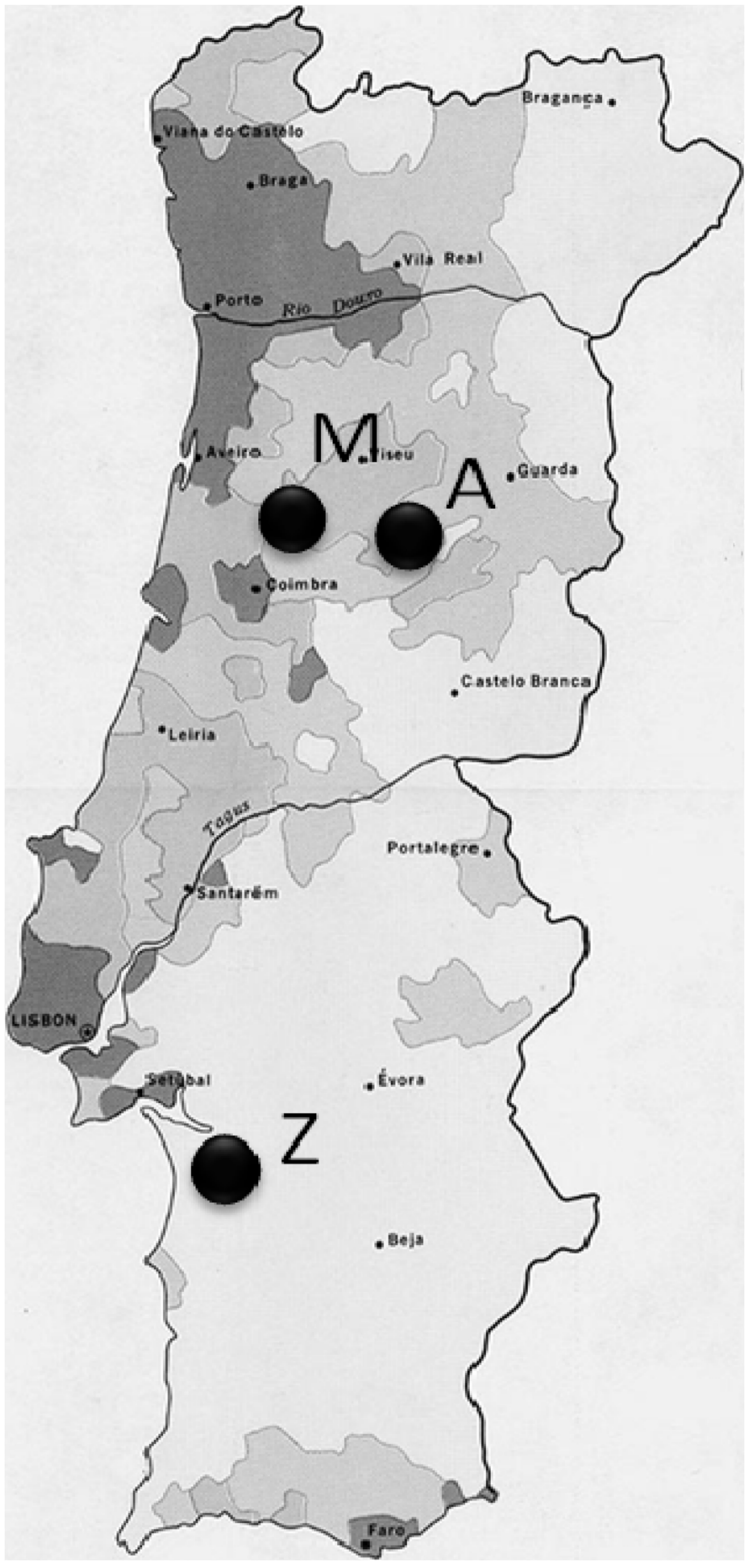
Sampling areas. Z - between Alcácer do Sal and Grândola, in the Setúbal District, M - Malhada Velha, Arganil and A - Avô, Oliveira do Hospital.

*Pinus pinaster* from three different areas affected by PWD in Portugal were sampled: one located between Alcácer do Sal and Grândola (Z), south Portugal (Setúbal District) and two other areas located in Coimbra District, Central Portugal, Malhada Velha, Arganil (M) and Avô, Oliveira do Hospital (A) (Fig. 1). The area Z is affected with PWD since 1999 and includes mainly *P. pinaster* and *P. pinea* trees with more than 30–35 years old. In this area, 4 symptomatic *P. pinaster* trees were sampled together with 4 asymptomatic *P. pinaster* trees. The areas M and A are affected with PWD since 2008 and include mainly *P. pinaster*, with a sparse number of *P. radiata*, *Quercus* and *Eucalyptus* trees. The area M included 109 trees: 11 *Quercus*, 7 *Eucalyptus*, 85 *P. pinaster* and 6 *P. radiata*. In this area, 12 *P. pinaste*r were symptomatic/dead and 2 *P. radiata* also showed symptoms. The sampling area A included a total of 116 *P. pinaster* and 15 *Quercus* trees where 18 *P. pinaster* were symptomatic. The presence of nematodes was screened in all symptomatic trees.

### Plant material

Sampling was performed in Spring-Summer. Each sample consisted of pinewood cross-sections from cut trees or wood obtained by drilling a 5 mm diameter hole to a depth of 10 to 15 cm with a sterilized hand brace drill (Haglof, Mora, Sweden). Fourteen *P. pinaster* trees were sampled at the trunk at breast height and at the top of the tree. The wood samples were placed in labelled and sealed individual plastic bags and divided in sub-samples for microbiological analysis and nematodes screening. All samples were kept at 4°C and analyzed within 24 h. The diameter of sampled trees was measured at breast height (DBH) and the trees classified into 6 symptom classes based on the symptoms they expressed: 0 – tree without symptoms, I - <10% brown leaves, II - 10–50% brown leaves, III – 50–80% brown leaves, IV - >80% brown leaves, V- dead tree without leaves.

### Nematode screening and identification

Nematodes were extracted, from triplicates of 20 g of each cross-section, using modified Baermann funnels [14]. After 48 h, the suspensions were collected and observed using an inverted stereomicroscope. The identification of PWN and other nematodes was based on the diagnostic morphological characters. *B. xylophilus* identification was confirmed molecularly by a satellite-DNA species-specific based technique [15].

### Microbial community associated with PWN and with other nematodes

The bark and sapwood of each sub-sample were removed under sterile conditions and the wood cut in ca. 2 cm chips. The wood pieces were placed in Petri dishes with R2A medium and incubated at 25°C, for three days. All bacterial colonies were isolated from the trails made by the nematodes on the medium. In order to isolate bacteria associated only with PWN, wood chips from infected wood samples from area Z were sterilized and processed as previously described and the bacterial colonies were selected only from the trails made by PWN on the medium. Molecular identification of PWN was performed using the satellite-DNA species-specific based technique [15].

Bacterial isolates were grouped by RAPD typing. RAPD fragments were amplified by PCR, using primer OPA-03 (5′ – AGT CAG CCA C – 3′) (Operon Technologies, Inc. Alameda, California, USA) together with crude cell lysates. DNA profiles for 123 isolates were grouped on basis of visual similarities of the fragments analyzed by electrophoresis in a 2% agarose gel stained with ethidium bromide. Reproducibility of the patterns was tested.

Nematodes were also extracted from infected wood samples from area Z, identified as *B. xylophilus* on the basis of morphological characters and sterilized with 0.1% sodium hypochlorite and washed with sterilized distilled water. The nematodes were then centrifuged and the resulting nematode pellet was homogenized with sterilized distilled water. DNA was extracted according to Nielsen et al.[16], the 16S rRNA gene from bacteria in the homogenate was amplified by PCR, cloned and sequenced as described below.

### 16S rRNA gene sequence of the bacterial isolates from nematode trails and nematode homogenates

Amplification of a nearly full-length 16S rRNA gene sequence from bacterial isolates and from nematodes homogenates was performed by PCR with primers 27F (5′ – GAG TTT GAT CCT GGC TCA G – 3′) and 1525R (5′ – AGA AAG GAG GTG ATC CAG CC – 3′) [17]. The PCR reaction mix (50 µl) contained: reaction buffer (1.5 mM MgCl_2_, 50 mM KCl and 10 mM Tris-HCl, pH 8.3), 100 µM (each) deoxynucleoside triphosphates (Promega, Madison, Wisconsin, USA), 0.2 µM (each) primer and 1.5 U Taq polymerase (Sigma, St. Louis, Missouri, USA). The PCR was performed with 30 cycles: 1 min at 94°C, 1 min at 55°C, and 1 min at 72°C.

PCR products with 1500 bp obtained from isolates were purified using the JET Quick PCR Purification Spin Kit (Genomed GmbH, Löhne, Germany) according to the manufacturer's instructions, and sequenced as described below. PCR products from nematode homogenates were cloned into pGEM-T Easy (Promega), transformed into *E. coli* XL1-Blue, extracted, amplified and purified according to standard procedures, and sequenced as described below.

### DNA sequence analyses

The 16S rRNA genes from 61 strains associated with nematodes trails and representing all RAPD groups and PCR products from nematode homogenates were subjected to amplification (or re-amplification in the case of the clones) for sequencing. Automated sequencing of the purified PCR products was performed using dRodamina terminator cycle-sequencing kit and ABI 310 DNA Sequencer (Applied Biosystems, Foster City, California, USA) according to the manufacturer's instructions.

### Phylogenetic analyses

All sequences were compared with sequences available in the EMBL/GenBank database using BLAST network services and with sequences in the Ribosomal Database Project II (RDP) [18]. Sequences were initially aligned with the CLUSTAL X program [19], visually examined, and relocated to allow maximal alignment. To obtain a more accurate phylogenetic assignment of the OTUs, the aligned sequences were divided into phyla and, in the case of *Proteobacteria*, into classes. Sequences were also checked for chimeric properties by using CHIMERA_CHECK program of RDP [18]. The method of Jukes and Cantor [20] was used to calculate evolutionary distances and phylogenetic dendrograms were constructed by the neighbor-joining method using the MEGA4 package [21].

### Siderophore production and proteolytic activity

All isolates were screened for their ability to produce siderophores when cultivated in CAS medium at 25°C during 48 h [22]. Strains developing an orange halo were considered as positive. The ability to degrade Tween 20, 40, 60 and 80 at concentration of 1.0% in R2A medium was tested after 3 and 5 days incubation at 25°C. Inoculated medium showing an opaque halo around the zone of growth was considered positive. Skim Milk Agar (R2A:skim milk, 1∶1, w/w) was used to detect proteolytic activity. Strains showing a transparent halo around the zone of growth were considered positive.

### Nucleotide sequence accession numbers

The 16S rRNA gene sequences of the isolates reported in this study have been deposited in EMBL database under the accession numbers from HQ538775 to HQ538819, from FJ784694 to FJ984701, FJ784703 from FJ784705 to FJ784710 and the cloned 16S rRNA genes sequences under the accession numbers FJ784711 and from FJ784713 to FJ784716.

## Results

### Nematode screening and identification

*Bursaphelenchus xylophilus* and other nematodes (Families *Rhabditidae* and *Aphelenchoididae*) were detected in 14 symptomatic trees (class III to V) from the 3 different affected areas, 4 from area Z, 5 from area M and 5 from area A (Fig. 1 and Table 1). Nematodes of family *Rhabditidae* and *Aphelenchoididae* were found in both symptomatic *P. radiata*.

**Table 1:** Sampled *Pinus pinaster* measured at breast height and classified based on the symptoms they expressed.

Area	*P. pinaster* tree(Code)	DBH(cm)	PWD symptom class 1)	*Bursaphelenchus xylophilus*	Other nematodes(Rhabditidae and Aphelenchoididae)
A(Oliveira do Hospital)	A12	12	III	+	+
	A25	18.5	III	+	+
	A37	24.5	V	+	+
	A38	23	III	+	+
	AB23	9.5	V	+	+
M(Arganil)	M24	28	V	+	+
	M47	29	V	+	+
	M67	14	V	+	+
	M68	11.5	V	-	+
	M72	8.5	V	-	+
Z(Grândola)	Z1	ND	ND	+	+
	Z2	ND	ND	-	+
	Z3	ND	ND	+	+
	Z5	ND	ND	+	+

ND  =  Not Determined.

DBH  =  Diameter Breast Height.

1) PWD symptom classes:

0 - tree without symptoms.

I - 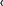 10% brown leaves.

II - 10–50% brown leaves.

III - 50–80% brown leaves.

IV - 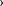 80% brown leaves.

V – dead tree without leaves.

### Microbial community associated with PWN and with other nematodes

**Figure 2:**
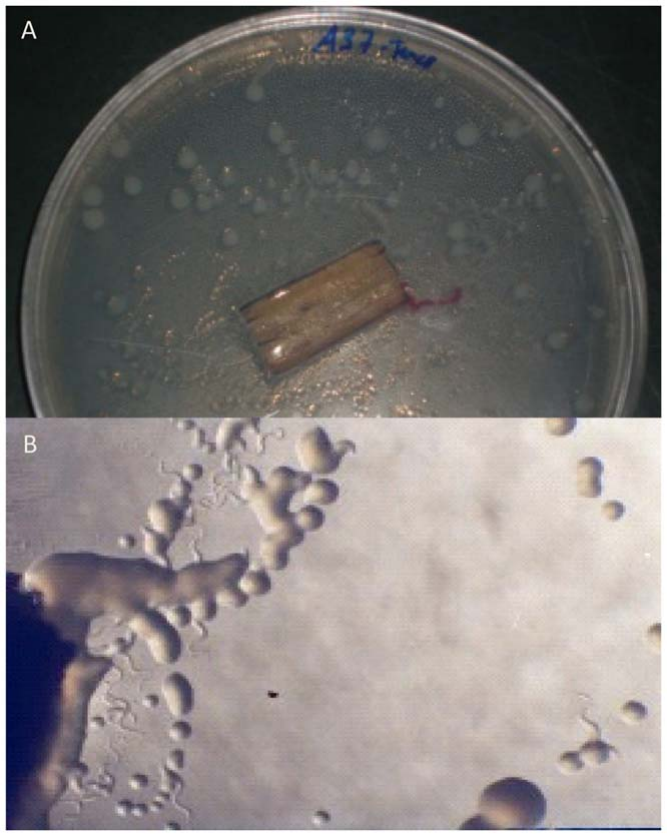
Bacterial colonies from nematode trails. Infected wood piece placed on Petri dish with R2A medium and incubated at 25°C, for three days (A). Bacterial colonies from the trails made by the nematodes on the medium were selected for characterization (B).

**Figure 3:**
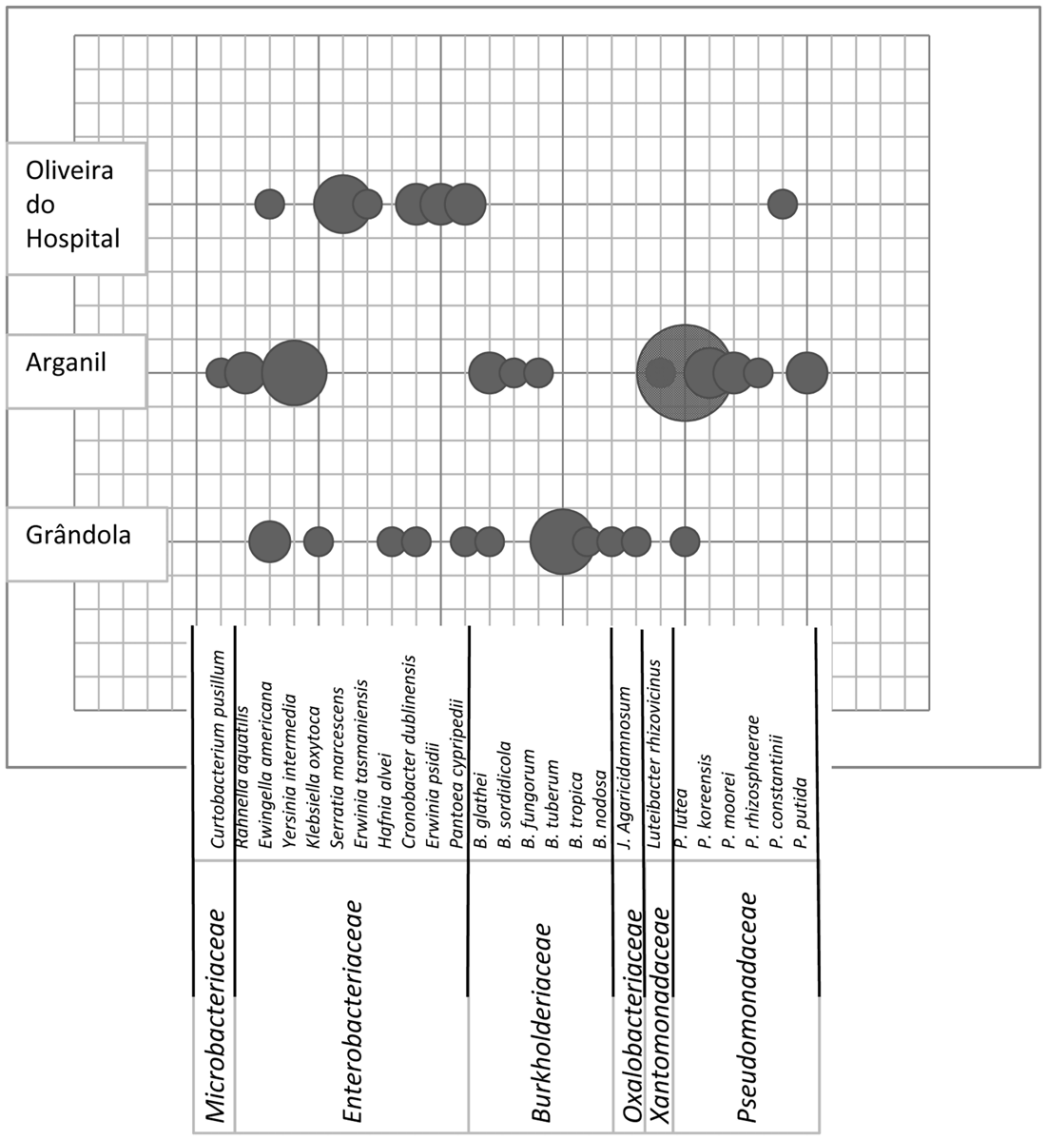
Site-dependent microbial diversity associated with PWN. Microbial community associated with the pinewood nematodes, isolated from the three forest areas (A-Oliveira do Hospital, M-Arganil and Z-Grândola) where Pine wilt disease was detected.

Nematode-associated bacteria were isolated from samples of 11 *P. pinaster* with PWN (Table 1). One hundred and twenty three strains were isolated from nematode trails (Fig. 2). The number of isolated bacteria and the species isolated were not related with the symptomatology or with the DBH of trees. These strains were grouped into 61 RAPD-types on basis of visual similarities. These strains belonged to two different phylogenetic groups: *Betaproteobacteria* and *Gammaproteobacteria* and one strain belonged to the *Actinobacteria* (family *Microbacteriaceae*) (Fig. 3). The strains of the *Betaproteobacteria* belonged to the genus *Burkholderia* (19.7%) and one strain was identified as *Janthinobacterium agaricidamnosum*, of the family *Oxalobacteriaceae* (ZR1-2). The isolates belonging to the *Gammaproteobacteria* were identified as strains of the family *Enterobacteriaceae* (41.0%), family *Pseudomonadaceae* (34.4%) and one strain was identified as *Luteibacter rhizovicinus* (*Xanthomonadaceae*) (M24-cE1).

**Figure 4:**
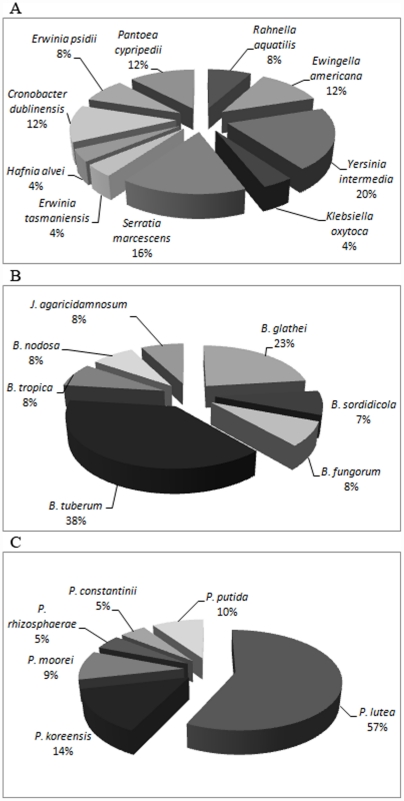
Microbial diversity of bacteria carried by *B. xylophilus* and other nematodes isolated in Portugal. Phylogenetic relationship of partial 16S rRNA gene sequences from isolated bacteria with selected reference sequences from identified bacteria in the database were obtained. A – *Gammaproteobacteria* classe - *Enterobacteriaceae* family; B – *Betaproteobacteria* classe *Burkholderiaceae* and *Oxalobacteriaceae* families; C - *Gammaproteobacteria* classe - *Pseudomonadaceae* family.

The family *Enterobacteriaceae* was represented by 10 different species (Fig. 4A). The most abundant species were *Yersinia intermedia* (20%) and *S. marcescens* (16%). The genera *Pantoea*, *Cronobacter*, *Erwinia* and *Ewingella* each included 12% of the isolates. The family *Burkholderiaceae* was represented by six species but the majority of the strains belonged to the species *B. tuberum* (38% of the *Betaproteobacteria*) (Fig. 4B). The second most abundant species was *B. glathei* (23%). The family *Pseudomonadaceae* was represented by 6 species with the most abundant species being *Pseudomonas lutea* (57%) (Fig. 4C).

The bacterial isolates associated only to *B. xylophilus* isolated from Grândola (area Z) were identified as belonging to the genus *Burkholderia* (50%) and to the *Enterobacteriaceae* (37.5%) and one strain was identified as *P. lutea*. One strain from the genus *Janthinobacterium* sp. (*Oxalobacteriaceae*) was isolated associated with PWN (Fig. 3).

Nematodes from Arganil trees (area M) carried bacteria from the genus *Pseudomonas* (59.4%), and *Burkholderia* (12.5%) and from the family *Enterobacteriaceae* (21.9%). The only *Luteibacter* and *Actinobacteria* strains were isolated in this area. In Oliveira do Hospital (area A), 92.3% of the strains associated with the nematodes belonged to the *Enterobacteriaceae* and only 7.7% to the genus *Pseudomonas*.

No single isolated bacterial species associated with *B. xylophilus* and other nematodes was common to all of the different forest areas samples. The *Enterobacteriaceae* associated with nematodes present in two different areas (M and A) were strains belonging to the species *Ewingella americana*, *Cronobacter dublinensis*, and *Pantoea cypripedii*. Strains from *Burkholderia glathei* and *P. lutea* were, respectively, the only *Burkholderiaceae* and *Pseudomonadaceae*, isolated from two areas (Z and M).

The clones from bacteria obtained from homogenates of disinfected PWN were identified as *Betaproteobacteria* belonging to the genus *Burkholderia* (N8) and *Janthinobacterium* (3-1.2), and as *Gammaproteobacteria* from family *Xantomonadaceae* belonging to the genus *Luteibacter* (strain Z4S-80 (2-1.4)) (Table 2). Two clones were identified as Gram-positive, an *Actinobacteria* belonging to the family *Corynebacteriaceae* (3-2.2) and a *Bacilli* belonging to the family *Streptococcaceae* (3-2.4).

**Table 2:** Assignment of taxonomic groups to uncultured bacterial clones from molecular nematode-associated bacteria clone libraries and the closest sequence match in database.

Clone	Bacterial family	Closest relative in database (EMBL accession no.)	Identity(%)	EMBL accession no.
N8	*Burkholderiaceae*	*Burkholderia tropica* strain TAt-0750 (EU723241)	99	FJ784711
2-1.4	*Xanthomonadaceae*	*Luteibacter* sp. Z4S-80 (FJ784633)	100	FJ784716
3-1.2	*Oxalobacteraceae*	Clone 14_H06 (FN421682)	100	FJ784713
3-2.2	*Corynebacteriaceae*	Clone nbw1098f01c1 (GQ054100)	100	FJ784714
3-2.4	*Streptococcaceae*	Clone ncd1053f11c1 (HM344016)	99	FJ784715

### Biochemical characterization

**Figure 5:**
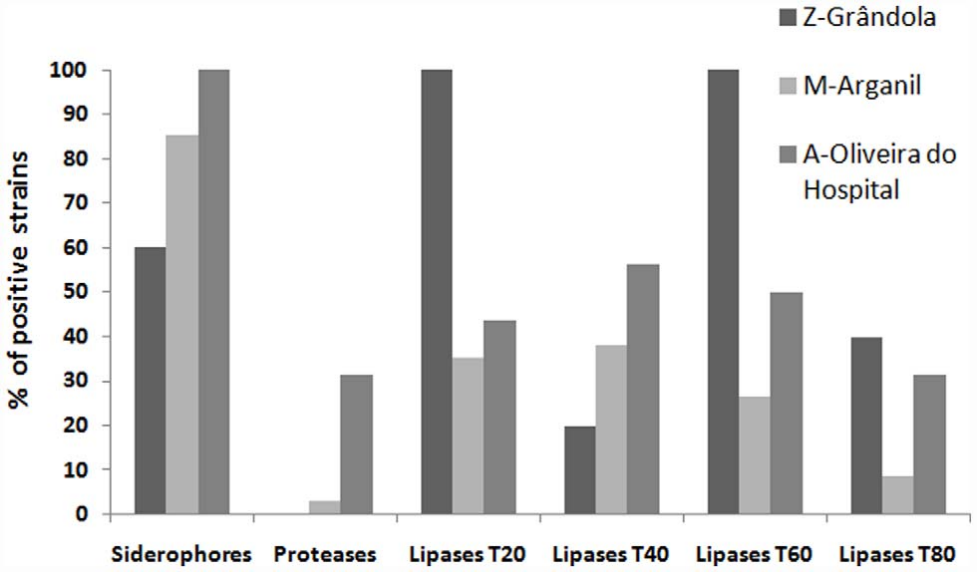
Production of siderophores and proteolytic activity of the bacteria isolated. Activity of strains isolated associated with nematodes from the 3 sampled areas (Z-Grândola, M-Arganil and A-Oliveira do Hospital).

All isolates were tested for lipases, proteases and siderophore production (Fig. 5). Most strains produced siderophores, from 60% of the isolates from Grândola (area Z) to 100% of the isolates from Oliveira do Hospital (area A). Proteases were produced only by the isolates from Oliveira do Hospital (30%) and by one isolate from Arganil (area M), but lipases were produced by 35 strains (63.6%) from the different forest areas. Lipase activity was best when using Tween 40 as substrate.

## Discussion

PWD has been detected in Portugal affecting *P. pinaster*. Therefore, it is important to understand first if the PWN isolated from infected trees carries bacteria and, second, what is the diversity of the bacteria associated with PWN and with other nematodes.

Previous works studied bacteria associated with PWN in the perspective that bacteria produce toxins that play an important role in PWD [23]. Bacteria producing toxins to both callus and the seedlings of Japanese black pine (*P. thunbergii*) were described. Zhao and Lin [11] described bacteria attached to the surface of PWN and Guo et al. [8] reported 209 as the average number of bacteria attached to the surface of PWN. Furthermore, authors claimed that healthy *P. thunbergii* did not possess bacteria in the tissues. This was against what was reported by Pirttilä et al. [24] which described the presence of bacteria in *P. sylvestris* tissues.

According to literature the isolates carried by PWN in different countries are mainly species of *Pseudomonas* (in China) and *Bacillus* (in Japan), with both genera in Korea [23]. Furthermore, the number of reported isolated bacterial strains associated with PWN and identified in the literature is low [25] and no information exists about differences in diversity of the bacteria carried by PWN or by other nematodes isolated from different forest zones.

In the present study, bacteria strains were isolated from *B. xylophilus* and other nematodes (Families *Rhabditidae* and *Aphelenchoididae*) isolated from *P. pinaster* with different symptom classes and different DBH. The number of isolated bacteria and the species isolated were not related with the symptomatology or with the DBH. The major phylogenetic groups of bacteria isolated associated with PWN and other nematodes present in trees with PWD were *Enterobacteriaceae* and strains from the genera *Burkholderia* and *Pseudomonas*. *Burkholderia* are reported for the first time associated with *B. xylophilus*, although strains from this genus are usually reported as endophytes. Additionally, Gram-positive bacteria associated with PWN were detected by molecular methods and one strain was isolated from nematode trails but none belonged to the genus *Bacillus*. Furthermore, the dominant populations were different according to the different areas. These differences could be explained by differences in the endophyte community of different tree species (from China, Japan, Korea and Portugal) and differences in the soil community. In this work, *Enterobacteriaceae* isolates belonged to different species but the most abundant was the species *Y. intermedia* (with one fifth of the strains), and species *S. marcescens*, *Pantoea cypripedii*, *Cronobacter dublinensis*, *Ewingella americana* and species belonging to the genus *Erwinia*. This phylogenetic group was reported to belong to the endophytic community of citrus, cocoa, eucalypti, soybean and sugar cane [26]. In fact, species *Erwinia tasmaniensis* and *Cronobacter dublinensis* were already reported as beneficial endophytes, while *Ewingella americana*, *Erwinia psidii* and *Pantoea cypripedii* are reported phytopathogens [27]. It is yet unclear if these *Enterobacteriaceae* are connected with the normal flora of *P. pinaster* or with PWD. Within the *Burkholderiaceae*, the species *B. tuberum* was the most common isolate but only carried by nematodes isolated from *P. pinaster* from Grândola (area Z). The presence of this species can most probably be related with its presence in the endophytic community of pine trees since *Burkholderia* species and its diversity were related with plant species and land use management [28]. The species of the genus *Pseudomonas* have been considered to be in mutualistic symbiosis with PWN and co-responsible for PWD [29]. Although species of the genus *Pseudomonas* were isolated from nematodes of all sites, the results of this study do not point to the possibility of a *Pseudomonas* species existing associated with PWN in Portugal, as none of the isolated *Pseudomonas* species was found in all 3 sampling areas. In fact, with the exception of *P. lutea*, which was isolated from nematodes of two different sampling areas, all the other isolated *Pseudomonas* species were different from area to area. Moreover, molecular techniques did not allow the detection of *Pseudomonas* in surface-disinfected nematodes. All species identified were described already associated with plants or from soil or even carried by insects. The type strain of *P. lutea* is able to solubilize phosphate [30] but *P. constantinii* is described as plant pathogenic bacteria [27]. Therefore additional research is needed in order to find out which is the role of these bacteria when associated to PWN and/or inside the plant.

The microbial community associated with Portuguese PWN and with other nematodes, produced siderophores and different lipases and did not produce proteases (casein hydrolysis). Plant lipases have been described as playing a role in plant defense [31]. Furthermore, the product of Tween 20 and 80 hydrolysis have been reported to be of essential importance in the regulation of a plant defense response [32]. Therefore, the lipase ability of the bacterial community could have a role, to be explained, in activation of plant defense.

This study contributes to the characterization of the diversity of the microbial community associated with *Bursaphelenchus xylophilus* and other nematodes present in *Pinus pinaster* with PWD. The majority of the strains isolated belonged to phylogenetic groups usually isolated as endophytic bacteria. Further work will be needed to understand the role of these bacteria in PWD.
